# Prognostic impact of bone marrow fibrosis in polycythemia vera: validation of the IWG-MRT study and additional observations

**DOI:** 10.1038/bcj.2017.17

**Published:** 2017-03-10

**Authors:** D Barraco, S Cerquozzi, C A Hanson, R P Ketterling, A Pardanani, N Gangat, A Tefferi

**Affiliations:** 1 Division of Hematology, Department of Medicine, Mayo Clinic, Rochester, MN, USA; 2 Department of Laboratory and Pathology, Mayo Clinic, Rochester, MN, USA

## Abstract

In 2012, the International Working Group for Myeloproliferative Neoplasms (MPN) Research and Treatment (IWG-MRT) reported an associations between mild bone marrow (BM) fibrosis (⩾grade 1) in polycythemia vera (PV) and a lower incidence of thrombosis during the clinical course and a higher risk of fibrotic progression. The objective in the current study of 262 patients with PV was to validate these observations and also identify other risk factors for myelofibrosis-free survival (MFFS). About 127 (48%) patients displayed ⩾grade 1 reticulin fibrosis at the time of diagnosis; presenting clinical and laboratory features were not significantly different between patients with or without BM fibrosis. In univariate analysis, BM fibrosis had no significant impact on overall, leukemia-free or thrombosis-free survival, whereas a significant association was noted for MFFS (*P*=0.009, hazard ratio 2.9; 95% confidence interval 1.32–6.78); other risk factors for MFFS included leukocytosis ⩾15 × 10^9^/l, presence of palpable splenomegaly and abnormal karyotype. During multivariable analysis, leukocytosis ⩾15 × 10^9^/l, palpable splenomegaly and ⩾grade 1 BM reticulin fibrosis remained significant. The current study validates the previously observed association between ⩾grade 1 BM reticulin fibrosis in PV and subsequent fibrotic progression, and identifies leukocytosis and palpable splenomegaly as additional risk factors for fibrotic progression; additional studies are required to clarify the impact of BM fibrosis on thrombosis and that of abnormal karyotype on MFFS.

## Introduction

Polycythemia Vera (PV) is a *BCR-ABL1* negative myeloproliferative neoplasm (MPN) characterized by clonal erythrocytosis and a *JAK2* mutation, either *JAK2V617F* or an exon 12 mutation, in 96% and 3% of cases, respectively.^1^ Life expectancy in PV has been shown to be worse than that of the age- and sex- matched US population, with a median survival of 14 years and 24 years in patients younger than age 60 years.^2^ Disease-related complications affecting survival in PV are thrombotic complications and disease progression into acute myeloid leukemia or myelofibrosis (MF).^3^ Numerous prognostic factors have been identified as being involved in clinical phenotype, disease progression and survival of PV patients. Tefferi *et al.*^2^ have identified leukocytosis, advanced age and venous thrombosis history as independent risk factors for overall survival (OS); myelofibrosis-free survival (MFFS) was reportedly affected by leukocytosis, older age, JAK2V617F allele burden, palpable splenomegaly, thrombocytosis and mild bone marrow (BM) fibrosis, whereas leukemia-free survival (LFS) was affected by advanced age, leukocytosis, abnormal karyotype, palpable splenomegaly and BM fibrosis.^2, 3, 4, 5, 6, 7^

As highlighted by the recently updated 2016 World Health Organization (WHO) classification of myeloid neoplasms and acute leukemia,^8^ specific histopathological BM features have a central role in the diagnosis of myeloproliferative neoplasms; furthermore, the degree of BM fibrosis might identify a more aggressive disease.^9, 10^ Increased BM reticulin fibrosis (⩾grade 1) in PV has been reported in 20%^11, 12^–51%^7^ of patients at time of diagnosis. In a previous report by the International Working Group for Myeloproliferative Neoplasms (MPN) Research and Treatment (IWG-MRT), mostly mild BM reticulin fibrosis (⩾grade 1 of a three-graded score system) at diagnosis was associated with a lower risk of thrombosis during the clinical course and a higher risk of fibrotic progression while it did not affect OS or LFS.^13^ The objectives for the current single-center study were to validate the observations from the IWG-MRT and also identify other risk factors for MFFS.

## Materials and methods

This study was approved by the institutional review board of Mayo Clinic (Rochester, MN). Study patients were selected from our institutional database of myeloproliferative neoplasm and fulfilled the 2016 WHO criteria for the diagnosis of PV.^8^ Clinical data collected were from the time of diagnosis. Cytogenetic analysis and reporting was done according to the International System for Human Cytogenetic Nomenclature.^14^ The degree of BM reticulin was based on 'real life' BM reports from Mayo Clinic hematopathologists and in accordance with the European consensus scoring system.^15^ Screening for the two most frequent and prognostically important mutations in PV^16^ other than *JAK2* (that is, *TET2* and *ASXL1*) were performed according to conventional methods.^17^ Differences in the distribution of continuous variables between categories were analyzed by either Mann–Whitney *U*-(for comparison of two groups) or Kruskal–Wallis test (comparison of three or more groups). Patient groups with nominal variables were compared by *χ*2-test. OS analysis was considered from the date of diagnosis to date of death (uncensored) or last contact (censored). MFFS, LFS and thrombosis-free survivals were determined from the time of diagnosis to the time the events occurrence after diagnosis (uncensored) or last contact/date of death (censored). All survival curves were prepared by the Kaplan–Meier methods and compared by the long-rank test. Cox proportional hazard regression model was applied to carry out multivariable analysis. *P*-values <0.05 were considered significant. The Stat View (SAS Institute, Cary, NC, USA) statistical package was used for all calculations.

## Results

Analysis was conducted on 262 patients who met 2016 WHO criteria of PV, and their clinical and laboratory characteristics are listed in Table 1. The median age was 62 years and 50% were males. Median values of hemoglobin, leukocytes and platelets were 18 g/dl, 11.7 × 10^9^/l and 454 × 10^9^/l, respectively. Among informative cases, palpable splenomegaly was present in 27%, pruritus in 33% and erythromelalgia in 6%. Thrombosis history at diagnosis was documented in 28% of the patients and 23% experienced thrombotic events after diagnosis. Mutational frequencies included, *JAK2* (*n* evaluable=258, 97%) *TET2* (*n* evaluable=80) 20% and *ASXL1* (*n* evaluable=80) 10%, respectively. Information on cytogenetic was available in 142 patients and karyotype was abnormal in 19%.

The cytogenetic abnormalities found at diagnosis were: isolated trisomy 9 (8 cases, 29%), loss of Y chromosome (5 cases, 18%), isolated trisomy 8 (5 cases, 19%), isolated deletion 20q (3 cases, 11%), isolated deletion 13q (2 cases, 7%), inversion of chromosome 1 (1 case, 4%), unbalanced translocation between chromosomes 9 and 18 (1 case, 4%), inversion of chromosome 2 (1 case, 4%) and concomitant presence of trisomy chromosome 9 and deletion 13q (1 case, 4%). BM reticulin fibrosis was reported to be absent in 135 patients (MF-0, 52%), grade 1 (MF-1) in 101 patients (39%), grade 2 (MF-2) in 22 patients (8%) and grade 3 (MF-3) in 4 (2%) patients. After a median follow up of 85 months, 107 (41%) deaths, 30 (11%) fibrotic progression and 5 (2%) leukemic transformations were documented.

The study population was subsequently stratified according to the presence (⩾grade 1 BM fibrosis) or absence of BM reticulin fibrosis. A number of clinical and laboratory parameters were evaluated for possible association with the presence of ⩾grade 1 BM reticulin fibrosis and none, including age (*P*=0.1), sex (*P*=0.7), hemoglobin level (*P*=0.9), leukocyte count (*P*=0.6), leukocytosis ⩾11 × 10^9^/l (*P*=0.6), leukocytosis ⩾15 × 10^9^/l (*P*=0.5), platelet count (*P*=0.4), presence of palpable splenomegaly (*P*=0.3), pruritus (*P*=0.5), erythromelalgia (*P*=0.7) or mutations (*JAK2*, *P*=0.2; *TET2*, *P*=0.4; *ASXL1*, *P*=0.3) displayed a significant association (Table 1).

**Figure 1:**
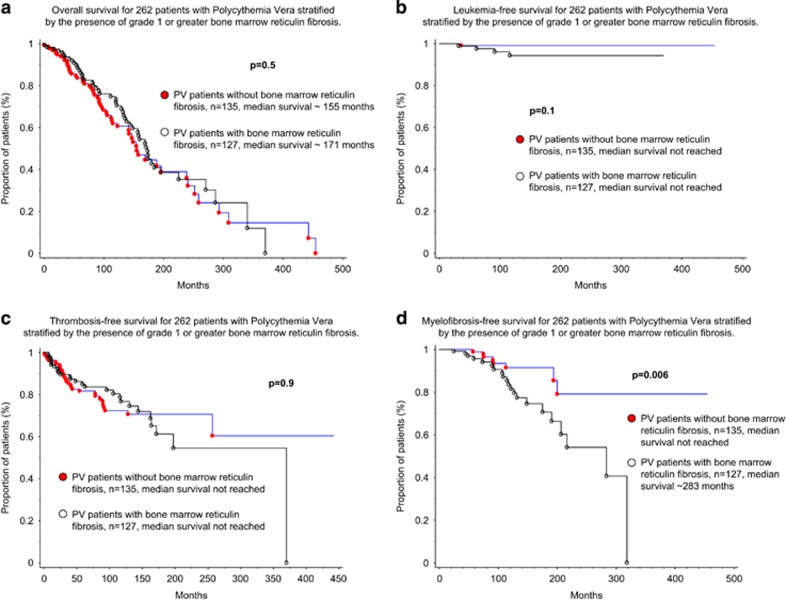
Kaplan–Meier curves representing OS (**a**), LFS (**b**), thrombosis-free survival (TFS) (**c**) and MFFS (**d**) in 262 patients with polycythemia vera stratified by the presence of grade 1 or greater bone marrow reticulin fibrosis (Table 2).

In univariate analysis, OS was adversely affected by age (as a continuous variable and as >60 years, *P*<0.0001 and *P*<0.0001, hazard ratio (HR) 5.1; 95% confidence interval (CI) 3.25–8.07, respectively), leukocytosis ⩾11 × 10^9^/l (*P*=0.03, HR 1.6; 95% CI 1.05–2.34) and ⩾15 × 10^9^/l (*P*=0.0001, HR 2.2; 95% CI 1.48–3.41) thrombosis history (*P*=0.005, HR 1.8; 95% CI 1.19–2.71) and the presence of *ASXL1* mutations (*P*=0.006, HR 3.6; 95% CI 1.43–8.97) but not by the presence of grade ⩾1 BM reticulin fibrosis (Figure 1a; *P*=0.5); in multivariate analysis, older age (>60 years), leukocytosis (⩾15 × 10^9^/l) and the presence of *ASXL1* mutation retained significance for OS (*P*<0.0001, *P*=0.001 and *P*=0.0006, respectively). LFS was negatively affected by the presence of palpable splenomegaly (*P*=0.03, HR 11.4; 95% CI 1.27–101.90) but not by BM reticulin fibrosis (Figure 1b; *P*=0.1); the latter did not affect thrombosis-free survival either (Figure 1c; *P*=0.9).

Patients with grade 1 or greater BM reticulin fibrosis were more prone to fibrotic progression (Figure 1d; *P*=0.006, HR 2.9; 95% CI 1.32–6.78). Other risk factors found to be prognostically significant for MFFS in univariate analysis were leukocytosis ⩾15x10^9^/l (*P*=0.02, HR 2.8; 95% CI 1.17–6.48), presence of palpable splenomegaly (*P*=0.02, HR 2.6; 95% CI 1.14–6.08) and abnormal karyotype (*P*=0.008, HR 6.3; 95% CI 1.62–24.84); on multivariable analysis, leukocytosis ⩾15 × 10^9^/l (*P*=0.04, HR 2.8; 95% CI 1.02–7.62), presence of splenomegaly (*P*=0.04, HR 2.4; 95% CI 1.02–5.8) and presence of BM reticulin fibrosis (*P*=0.02, HR 3.3; 95% CI 1.24–8.60) remained significant (Table 2). Presence of abnormal karyotype was significant in univariate analysis for MFFS (*P*=0.008, HR 6.3; 95% CI 1.62–24.84) and was of borderline significance during multivariable analysis (*P*=0.06); a similar trend of significance was noted between abnormal karyotype and LFS (*P*=0.05; data not shown).

## Discussion

The IWG-MRT previously evaluated the prognostic significance of grade ⩾1 BM fibrosis in 526 patients with PV and reported on the impact of BM fibrosis on thrombosis-free survival (favorable) and MFFS (unfavorable), without affecting OS or LFS.^13^ In the current study, we sought to validate these previous observations and identify additional risk factors for MFFS. The two studies (the aforementioned IWG-MRT and the current study) were concordant in their observations regarding the lack of associations with OS and LFS, and the unfavorable impact of BM fibrosis on MFFS; the discrepancy on the association with thrombosis-free survival requires additional studies for clarification. Regardless, it is not appropriate to directly compare the two studies as the subjective criteria for BM fibrosis might have been different; for example, the incidence of grade ⩾1 BM fibrosis in the current study was much higher than the one reported by the IWG-MRT study (48% vs 14%, respectively) and we reported a higher proportion of cases with higher degree of fibrosis (MF-2 and MF-3, 26 vs 2 cases, respectively). In this regard, it is important to note that our study populations with or without BM fibrosis were similar in their presenting clinical and laboratory features (Table 1).

In both the current study and that of the IWG-MRT, the presence of mild BM fibrosis did not affect OS or LFS; in the current study, risk factors for OS or LFS included advanced age (⩾60 years), leukocytosis (⩾15 × 10^9^/l), *ASXL1* mutations and palpable splenomegaly, and these observations were consistent with previous reports.^2, 7, 13, 18^ The current study also shows the limited prognostic relevance of *ASXL1* or *TET2* mutations for MFFS, whereas a possible impact from abnormal karyotype was suggested despite the lower number of informative cases; the latter has previously been shown to affect both OS and LFS in PV.^2, 18, 19^

The current study highlights the overall prognostic value of including BM examination during the diagnostic evaluation of PV, by demonstrating associations between fibrotic progression and the presence of mild reticulin fibrosis or abnormal karyotype at the time of diagnosis. Additional validation studies are required to confirm the association between fibrotic progression and abnormal karyotype, and resolve the discrepancy between the current study and that of the IWG-MRT, in terms of the association between BM fibrosis and risk of thrombosis.

## Figures and Tables

**Table 1:** Presenting clinical and laboratory features of 262 patients with polycythemia vera stratified by the presence of grade 1 or greater bone marrow reticulin fibrosis

*Variables*	*All patients (*n*=262)*	*Patient without BM reticulin fibrosis (*n*=135, 52%)*	*Patients with*⩾*grade 1 BM reticulin fibrosis (*n*=127, 48%)*	P-*value*
Age at referal in years; median (range)	62 (17–94)	64 (20–88)	60 (17–94)	0.08
Age⩾60, *n* (%)	141 (54%)	79 (59%)	62 (49%)	0.1
Male (%)	131 (50%)	69 (51%)	62 (49%)	0.7
Hemoglobin, g/dl median (range)	18 (14.8–24)	18 (15.8–23)	18 (14.8–24)	0.9
Leukocytes, × 10^9^/l; median (range)	11.7 (4.3–59.3)	11.7 (4.7–59.3)	11.5 (4.3–35.5)	0.6
Leukocytes⩾11x10^9^/l; median (range)	142 (57%)	73 (56%)	69 (59%)	0.6
Leukocytes⩾15x10^9^/l; median (range)	58 (23%)	33 (25%)	25 (21%)	0.5
Platelets, × 10^9^/l; median (range)	454 (44–2747)	456 (78–1720)	450 (44–2747)	0.4
Platelets⩾1.000 x10^9^/l; median (range)	14 (6%)	9 (7%)	5 (4%)	0.3
Presence of palpable splenomegaly*n* (%) *N* evaluable=233	60 (26%)	28 (23%)	32 (29%)	0.3
Presence of pruritus *n* (%) *N* evaluable=253	83 (33%)	40 (31%)	43 (35%)	0.5
Presence of erythromelagia *n* (%) *N* evaluable=226	14 (6%)	6 (6%)	8 (7%)	0.7
				
*WHO PV risk stratification*				0.07
Low risk	81 (31%)	35 (26%)	46 (36%)	
High risk	181 (69%)	100 (74%)	81 (64%)	
				
Thrombosis history at diagnosis	74 (28%)	40 (30%)	34 (27%)	0.6
Thrombotic events after diagnosis	59 (23%)	31 (23%)	28 (22%)	0.9
				
*Cytogenetics categories* N *evaluable=142*				
Normal cytogenetic	115 (81%)	64 (83%)	51 (78%)	0.5
Favorable cytogenetic^a^	136 (96%)	73 (95%)	63 (97%)	0.5
				
*JAK2* *n* (%) *N* evaluable=258	250 (97%)	128 (96%)	122 (98%)	0.2
*TET2* *n* (%) *N* evaluable=80	16 (20%)	11 (23%)	5 (15%)	0.4
*ASXL1* *n* (%) *N* evaluable=80	8 (10%)	6 (13%)	2 (6%)	0.3

Abbreviations: BM, bone marrow; PV, polycythemia vera; WHO, World Health Organization.

a Favorable karyotype: normal karyotype or sole or two abnormalities that do not include the below-listed unfavorable cytogenetic abnormalities. Unfavorable karyotype: complex karyotype or sole or two abnormalities that include +8, 7/7q-, i(17q), 5/5q-, 12p-, inv(3) or 11q23 rearrangement.

**Table 2:** Univariate and multivariate analysis of prognostic factors for overall, myelofibrosis and leukemia-free survivals in patients with polycythemia vera (*n*=262)

*Variables*	*Overall survival*		*Myelofibrosis-free survival*		*Leukemia-free survival*	
	*Univariate analysis*	*Multivariate analysis*	*Univariate analysis*	*Multivariate analysis*	*Univariate analysis*	*Multivariate analysis*
BM reticulin fibrosis⩾grade 1	*P*=0.5		***P***=**0.009** **HR 2.9; 95% CI 1.32–6.78**	***P***=**0.02** **HR 3.3; 95% CI 1.24–8.60**	*P*=0.2	NA
Age	***P***<0**.0001**		*P*=0.4		*P*=0.7	NA
Sex	*P*=0.2		*P*=0.6		*P*=0.6	NA
Age>60 years	***P***<**0.0001** **HR 5.1; 95% CI 3.25–8.07**	***P***<0**.0001** **HR 6.1; 95% CI 2.66–13.96**	*P*=0.4		*P*=0.8	NA
Hemoglobin, g/dl	*P*=0.6		*P*=0.3		*P*=0.6	NA
Platelets, × 10^9^/l	*P*=0.2		*P*=0.8		*P*=0.08	NA
WBC × 10^9^/l	***P***=**0.0002**		*P*=0.1		*P*=0.9	NA
WBC⩾11 × 10^9^/l	***P***=**0.03** **HR 1.6; 95% CI 1.05–2.34**		*P*=0.4		*P*=0.7	NA
WBC⩾15 × 10^9^/l	***P***=**0.0001** **HR 2.2; 95% CI 1.48–3.41**	***P***=0**.001** **HR 3.3; 95% CI 1.60–6.93**	***P***=**0.02** **HR 2.8; 95% CI 1.17–6.48**	***P***=**0.04** **HR 2.8; 95% CI 1.02–7.62**	*P*=0.2	NA
Palpable splenomegaly at diagnosis	*P*=0.1		***P***=0**.02** **HR 2.6; 95% CI 1.14–6.08**	***P***=**0.04** **HR 2.4; 95% CI 1.02–5.8**	***P***=**0.03** **HR 11.4; 95% CI 1.27–101.90**	NA
Presence of pruritus	*P*=0.6		*P*=0.09		*P*=0.3	NA
Presence of erythromelagia	*P*=0.8		*P*=0.8		*P*=0.2	NA
Thrombosis history	***P***=**0.005** **HR 1.8; 95% CI 1.19–2.71**	*P*=0.2	*P*=0.5		NA	
						
*Thrombotic events after diagnosis*	*P*=0.9		*P*=0.7		NA	NA
* JAK2*	*P*=0.47		*P*=0.6		NA	
* TET2*	*P*=0.2		*P*=0.8		NA	
* ASXL1*	***P***=**0.006** **HR 3.6; 95% CI 1.43–8.97**	***P***=0**.0006** **HR 5.4; 95% CI 2.05–14.06**	*P*=0.4		*P*=0.1	NA

Abbreviations: BM, bone marrow; CI, confidence interval; HR, hazard ratio; NA, not applicable; WBC, white blood cell count. Bold font indicates significant *P*-values.
